# Sex differences in gastrointestinal permeability during acute uncompensable heat stress: Role of menstrual phase hormones

**DOI:** 10.1113/EP094141

**Published:** 2026-11-15

**Authors:** Shoji Leach, Ellie J. Allsop, Isobel A. Martin, Thomas Francis, Stephen D. R. Harridge, Tessa R. Flood, Zachary J. McKenna, John M. Halket, Anna Caldwell, Nabeel Sheikh, Ana Rodriguez‐Mateos, Josh Foster

**Affiliations:** ^1^ Centre for Human and Applied Physiological Sciences Faculty of Life Sciences and Medicine King's College London London UK; ^2^ Department of Psychological and Sport Sciences University of West London London UK; ^3^ Department of Health, Human Performance and Recreation University of Arkansas Fayetteville Arkansas USA; ^4^ Department of Nutritional Sciences School of Life Course and Population Health Sciences Faculty of Life Sciences and Medicine King's College London London UK; ^5^ Cardiothoracic Centre Guy's and St Thomas’ Hospital London UK

**Keywords:** gastrointestinal permeability, heat stress, hyperthermia, menstrual cycle, oestradiol, sex, uncompensable heat

## Abstract

Hyperthermia can increase gastrointestinal permeability, allowing microbes to leak from the intestinal lumen to the hepatic portal and systemic circulation. However, the extent to which sex and menstrual cycle hormones impact the risk of increased gastrointestinal permeability during heat stress is unclear. Twenty‐four healthy young adults [12 male, 24 ± 3 years old; 12 female (5 oral contraceptive users), 23 ± 2 years old] participated in the study. Initially, participants completed a control trial (no heat or exercise) to determine baseline gastrointestinal permeability. Then, participants took part in a hyperthermia trial, in which deep body temperature was increased by +1.5°C from baseline via a water‐perfused suit combined with semi‐recumbent cycling at 40 W. Oestradiol was measured at baseline during the hyperthermia trial. In both trials, permeability was assessed by measuring the urinary excretion of orally ingested sucrose (10 g) and the lactulose/rhamnose ratio (L/R, 1 g each) to assess gastroduodenal and small intestinal permeability, respectively. Data are presented as the change in excretion from control to hyperthermia. Pooled analysis of all participants identified that hyperthermia increased small intestinal permeability [ΔL/R = 0.012 ± 0.029 (95% confidence interval 0.000 to 0.025), *P* = 0.050, $d_{z}$ = 0.43]. Although there were no sex differences at the group level, we identified a strong positive correlation between ΔL/R and baseline oestradiol concentration within females (*r* = 0.709, *P* = 0.040). Gastroduodenal permeability did not increase with hyperthermia (*P* = 0.136), nor were there any differences between the sexes (*P* = 0.411). Future studies should confirm whether oestradiol plays an independent role in modulating heat‐induced gastrointestinal permeability in humans.

## Abstract (highlights)

- **What is the central question of this study?**
  Are there sex differences in gastrointestinal permeability during uncompensable heat stress, and are responses modulated by menstrual cycle hormones?
- **What is the main finding and its importance?**
  Small intestinal permeability increased in response to uncompensable heat stress, but there were no sex differences at the group level. However, there was a strong positive correlation between small intestinal permeability and baseline oestradiol concentrations within females. Future research should confirm whether oestradiol plays an independent role in modulating heat‐induced gastrointestinal permeability in humans.

## 1 INTRODUCTION

Workers in specialised occupations (e.g., firefighters, military, hazardous waste disposal) are at high risk of uncompensable heat stress (UHS), a state in which heat gain into the body cannot be offset equally to the environment, causing body temperature to rise continuously until collapse or removal from those conditions (Cheung et al., 2000). UHS is prevalent across a number of occupational heat stress situations, particularly those involving combinations of ambient heat, protective clothing and moderate to high work rates (Cheung et al., 2000). Furthermore, the prevalence of UHS at the population level is increasing with climate change, resulting from simultaneous combinations of high temperature and humidity (Powis et al., 2023; Vecellio et al., 2023).

Females are increasingly represented in heat‐vulnerable occupations, including firefighting, construction and the armed forces. In the UK, female representation across these sectors has increased by ∼5% in the last decade (Ministry of Defence, 2025; Ministry of Housing, 2025). Furthermore, healthcare‐related occupations, such as nursing, already have a large female representation and often combine highly impermeable personal protective equipment with indoor overheating. For example, the COVID‐19 pandemic was met with frequent heat complaints from nurses (a higher occurrence in females than in males), which likely impacted their ability to work safely and effectively (Bongers et al., 2022; Jegodka et al., 2021). However, despite a large female representation in industries at risk of UHS, the impact of sex per se on end‐organ injury during UHS remains poorly understood. At a population level, factors that could increase heat‐related risks in females could stem from body characteristics (lower body mass and higher percentage fat) (Havenith et al., 1995), lower aerobic fitness (Havenith & van Middendorp, 1990), lower maximum sweat gland output (Gagnon & Kenny, 2012) and varying menstrual cycle hormones (Charkoudian & Stachenfeld, 2014).

Although UHS markedly increases thermal and cardiovascular stress (Duncan et al., 1979; White et al., 1991), little is known about the downstream impacts on gastrointestinal permeability. In normal conditions, the gastrointestinal barrier prevents the leakage of microbes (bacteria, fungi and viruses) from the intestinal lumen into the systemic circulation (Hooper & MacPherson, 2010). Therefore, heat‐induced damage to this barrier could facilitate microbial translocation, leading to heatstroke/sepsis in extreme cases (Leon & Helwig, 2010). In occupational settings, gastrointestinal barrier damage induced by extreme heat could lead to similar downstream health effects.

The gold standard for measuring gastrointestinal barrier permeability is the multi‐sugar recovery method. Briefly, the extent to which an orally administered sugar type is recovered in the urine indicates the level of regional gastrointestinal permeability. For example, the lactulose/rhamnose (L/R) ratio and sucrose recovery are indicators of small intestine and gastroduodenal permeability, respectively (van Wijck et al., 2012). Heat stress has repeatedly been shown to increase gastrointestinal permeability (Houghton et al., 2023; McKenna et al., 2024; Pugh et al., 2017; Rubio et al., 2021; Selkirk et al., 2008; Zuhl et al., 2015), secondary to non‐cutaneous vasoconstriction in the splanchnic vascular beds (Minson et al., 1998; Rowell et al., 1971). However, only limited data are available to determine whether sex differences in such responses exist. Rubio et al. (2021) demonstrated reduced small intestinal permeability in females during prolonged intermittent sauna exposure, and Snipe and Costa (2018) showed similar increases in gastrointestinal permeability between sexes in response to 2 h of running in the heat at 60% of maximal oxygen consumption (${\dot{V}}_{O_{2}\max}$). Animal studies have shown that menstrual phase hormones (oestradiol and progesterone) might play key regulatory roles in gastrointestinal permeability. For example, in mice subjected to heatstroke, ovariectomy potentiated gastrointestinal barrier damage, leading to an accelerated onset of collapse (Rentería et al., 2023). However, the independent effects of oestradiol and progesterone were not isolated, making it challenging to understand the individual roles of each hormone. Understanding whether menstrual cycle hormones modulate heat‐induced gastrointestinal permeability in humans might better inform safety standards during occupational heat stress.

Therefore, the primary aim of the study was to investigate the effects of sex on gastrointestinal permeability during short‐term UHS. A secondary aim was to investigate whether any changes in such markers were associated with the menstrual cycle hormones oestradiol and progesterone.

## 2 MATERIALS AND METHODS

### 2.1 Participants

Twenty‐four young adults (21–33 years of age) participated in the study (Table 1). The study was approved by King's College London Research Ethics Committee (HR/DP‐23/24‐42188), which conformed to the standards set out in accordance with the *Declaration of Helsinki*. Signed informed consent was provided by all participants before participation. Exclusion criteria included: (1) any chronic medical conditions requiring regular medication/therapy, such as cancer, diabetes, uncontrolled hypertension and uncontrolled hypercholesterolaemia; (2) abnormalities detected on routine ECG screening suggestive of provocable ischaemia, undetected cardiac disease or resting left bundle branch block; (3) current smoker or regularly smoked within the past 3 years; (4) body mass index ≥ 35 kg/m^2^; (5) known gastrointestinal issues, such as inflammatory bowel disease, Crohn's disease, ulcerative colitis or previous abdominal surgery; (6) pregnancy (confirmed in young females using a urine pregnancy test); (7) diagnosed psychiatric illness or disease; or (8) previous history of diagnosed heatstroke.

**TABLE 1:** Participant characteristics.

Characteristic	Males	Females	*P*‐value
Age, years	24 ± 3	23 ± 2	0.318
Height, cm	175 (11)	166 (9)	0.001^*^
Body mass, kg	83.9 ± 13.7	60.8 ± 6.7	<0.001^*^
BMI, kg/m^2^	26 ± 4	22 ± 2	0.003^*^
BSA, m^2^	2.02 ± 0.18	1.68 ± 0.11	<0.001^*^
MSSA, cm/kg	244 ± 21	277 ± 14	<0.001^*^
Baseline SBP, mmHg	121 ± 8	109 ± 6	<0.001^*^
Baseline DBP, mmHg	77 ± 5	70 ± 8	0.023^*^
Baseline MAP, mmHg	91 ± 4	82 ± 8	0.001^*^
${\dot{V}}_{O_{2}\max}$, mL/min/kg	46.9 (7.1)	47.2 (13.4)	0.514
OC user	–	5	–

*Note*: Values are presented as either the mean ± SD or the median (interquartile range). Males, *n* = 12. Females, *n* = 12.

Abbreviations: BMI, body mass index; BSA, body surface area; DBP, diastolic blood pressure; MAP, mean arterial pressure; MSSA, mass‐specific surface area; OC, oral contraceptive; SBP, systolic blood pressure; ${\dot{V}}_{O_{2}\max}$, maximal oxygen consumption.

*Significant difference between males and females (*P* < 0.05).

### 2.2 Preliminary testing

Participants completed a screening visit, which involved a medical history form, a physical activity questionnaire, and a heat exposure questionnaire. In addition, we measured height (Seca 213, Seca, USA), body mass (TFW 150, Adam Equipment, UK), body composition (Marsden MBF‐6010, Marsden, UK), heart rate and rhythm via 12‐lead ECG (MAC 600, GE HealthCare, USA), blood pressure (Tango M2, SunTech, USA) and plasma cholesterol (CardioChek Professional Analyser, BHR Pharmaceuticals, UK) via a fingerprick blood sample. If participants passed the screening, they took part in a multistage submaximal exercise test on a cycle ergometer (Excalibur Sport, Lode, The Netherlands) to estimate ${\dot{V}}_{O_{2}\max}$. A submaximal test was conducted in favour of a maximal test for safety purposes, because the present study was part of a bigger research project including older participants. The submaximal test used the Young Men's Christian Association (YMCA) protocol as described by Golding and Sinning (1989). Estimated ${\dot{V}}_{O_{2}\max}$ was calculated by extrapolating the linear regression between heart rate and ${\dot{V}}_{O_{2}}$ with the predicted heart rate maximum (220 minus age).

### 2.3 Study design and experimental controls

Participants arrived at the Centre for Human and Applied Physiological Sciences, King's College London on two occasions. Trial order was fixed such that the first visit consisted of a thermoneutral control trial, and the second visit was a controlled hyperthermia trial. This approach was chosen to familiarise participants gradually to a new laboratory setting and procedures, reducing anxiety during the heat visit, which could have skewed the permeability results. Participants provided written confirmation that they had not used corticosteroids within the previous 6 weeks, antibiotics within the previous 4 weeks, or non‐steroidal anti‐inflammatory drugs within the previous week, to minimise potential effects on gastrointestinal barrier integrity and inflammatory responses. Furthermore, they confirmed no use of supplements known to influence gastrointestinal barrier function (i.e., probiotics, glutamine, bovine colostrum, etc.). Participants were instructed to arrive at the laboratory between 08.00 and 09.00 h to minimise the effects of diurnal variations between participants. Participants were asked to refrain from alcohol consumption, planned exercise and spicy food for 24 h, and withheld from caffeine for ≥12 h to minimise potential effects on gastrointestinal barrier integrity (Camilleri, 2021). They were instructed to fast overnight for 12 h prior to arrival and to arrive hydrated for all laboratory visits. To confirm adherence to these nutritional controls, participants recorded a food diary for 24 h preceding each visit. Upon arrival on each visit, participants provided a urine sample to measure urine specific gravity (OPTi digital handheld refractometer, Bellingham + Stanley, Germany) to confirm euhydration (urine specific gravity ≤ 1.025). Use of oral contraception and day of last menstrual cycle period were reported by females during both visits. Visits were not deliberately scheduled within the same menstrual cycle phase. Visits were separated by ≥48 h.

### 2.4 Multi‐sugar solution to assess gastrointestinal permeability

Participants ingested a multi‐sugar solution dissolved in 100 mL of water in each trial to assess gastroduodenal and small intestinal permeability, based on published protocols (McKenna et al., 2024; Rubio et al., 2021). Gastroduodenal permeability was assessed by ingesting 10 g of sucrose (APC Pure, UK), and small intestinal permeability was assessed by ingestion of 1 g each of lactulose (Esteve Pharmaceuticals, Germany) and l‐rhamnose (Sigma–Aldrich, USA). The ratio of lactulose/rhamnose is used to control for variation in gastrointestinal motility and kidney function, because rhamnose freely crosses the intestinal epithelium through transcellular mechanisms, whereas lactulose can cross only through paracellular mechanisms (König et al., 2016). Participants collected urine for 3 h after sugar ingestion in a 3 L plastic container, which was then homogenised and aliquoted into 3 × 1.5 mL Eppendorf tubes. Samples were centrifuged at 21 000*g* for 25 min at 4°C, after which 1 mL of each supernatant was stored at −80°C for later analyses.

### 2.5 Control visit

Once euhydration and adherence to experimental controls were confirmed, participants ingested a multi‐sugar solution (described in section 2.4) dissolved in 100 mL of water. This was completed in thermoneutral conditions as a determination of baseline gastrointestinal permeability with no confounding effect of heat or exercise. For the following 3 h post sugar ingestion, participants then self‐collected urine in a 3 L plastic container. During this period, participants remained fasted and rested. The storage and analysis of the sugars are described in detail in section 2.8.

### 2.6 Hyperthermia visit

Upon arrival, euhydration was confirmed via urine sample analysis, and participants confirmed adherence to experimental controls. Gastrointestinal temperature was used as an index of deep core temperature (*T*_core_) and was measured continuously via oral ingestion of a telemetric pill (eCelsius Medical, BodyCAP, France) ingested ≥1 h prior to baseline, in line with published recommendations (Notley et al., 2021). Prior to equipment instrumentation and immediately post heating, a nude body mass (TFW 150, Adam Equipment, UK) was measured to calculate whole‐body sweat loss. Skin thermistors (AD Instruments, New Zealand) were attached to four sites on the right side of the participant (triceps, pectoral, quadriceps and calf) using adhesive tape. Individual skin temperature data were input into the four‐parameter Ramanathan equation to determine mean skin temperature (*T*_sk_) (Ramanathan, 1964). Heart rate was measured via a three‐lead ECG (LP10G, HME LifePulse, UK), and exercise brachial blood pressure was recorded using automated ECG‐gated brachial oscillometry (Tango M2, SunTech, USA) at 15 min intervals. In addition, a laser Doppler flowmeter (moorVMS‐LDF2, Moor Instruments Ltd, UK) attached to a local heater (moorLAB‐Heat, Moor Instruments Ltd, UK), was placed on the right forearm to measure an index of skin blood flow. Laser Doppler probes were calibrated by placing the probes in a flux standard (PFS, Moor Instruments Ltd, UK). Cutaneous vascular conductance (CVC) was calculated by dividing laser Doppler flux by mean arterial pressure (MAP), expressed as flux per millimetres of mercury. Local heating was performed for 20 min at the end of the trial to provide a maximal flux value, allowing CVC to be presented as a percentage of maximum. Perceptual measures were recorded in 10 min intervals, including rating of perceived exertion (RPE; 15‐point scale: 6 = no exertion, 20 = maximal exertion; Borg, 1970), thermal comfort (7‐point scale: 1 = comfortable, 7 = very uncomfortable) and thermal sensation (100‐point scale: −50 = extremely cold, 0 = neutral, 50 = extremely hot). The descriptors for each of these scales comply with international standards for measuring thermal comfort and sensation (ISO10551, 2001). An analog‐to‐digital converter (Powerlab 16sP, ADInstruments, New Zealand) continuously collected and stored heart rate, *T*_sk_ and forearm skin blood flow data. Following instrumentation, participants donned a water‐perfused tube‐lined suit (Med‐Eng, Canada) covering their entire body except for the head, hands and feet.

Participants then rested supine for 15 min of baseline whilst thermoneutral water (35°C) circulated through the suit from a water cooler (ECOCOOL 150R, Grant Instruments, UK). Following baseline, participants sat on a recumbent cycle ergometer (Angio, Lode, The Netherlands) and began cycling at 40 W while the temperature circulating through the water‐perfused suit was increased, then maintained at 50°C (Figure 1). Additionally, a short‐sleeved thermal top, leggings, and a plastic sheet were placed over the participant to minimise the potential for heat loss and therefore accelerate heat gain. When *T*_core_ increased by 0.5°C from baseline, participants ingested the multi‐sugar solution dissolved in 100 mL of water. The multi‐sugar solution was ingested at this point in the trial to ensure that gastrointestinal permeability was assessed during a period of high thermal strain (McKenna et al., 2024). The controlled hyperthermia phase of the trial continued until *T*_core_ increased by 1.5°C from baseline. Other termination criteria included a *T*_core_ of 39.5°C, symptoms of nausea, dizziness or light‐headedness or voluntary participant withdrawal for any other reason; however, all participants continued the protocol until the 1.5°C termination criterion was met. Once an increase of 1.5°C was reached, the local heater on the participant's forearm was turned on to 45°C for 20 min to elicit a maximum skin blood flow response. After this, participants remained rested and fasted; they were encouraged to drink water, and they collected urine produced in a 3 L container for 3 h following the time point when the multi‐sugar solution was ingested.

**FIGURE 1:**
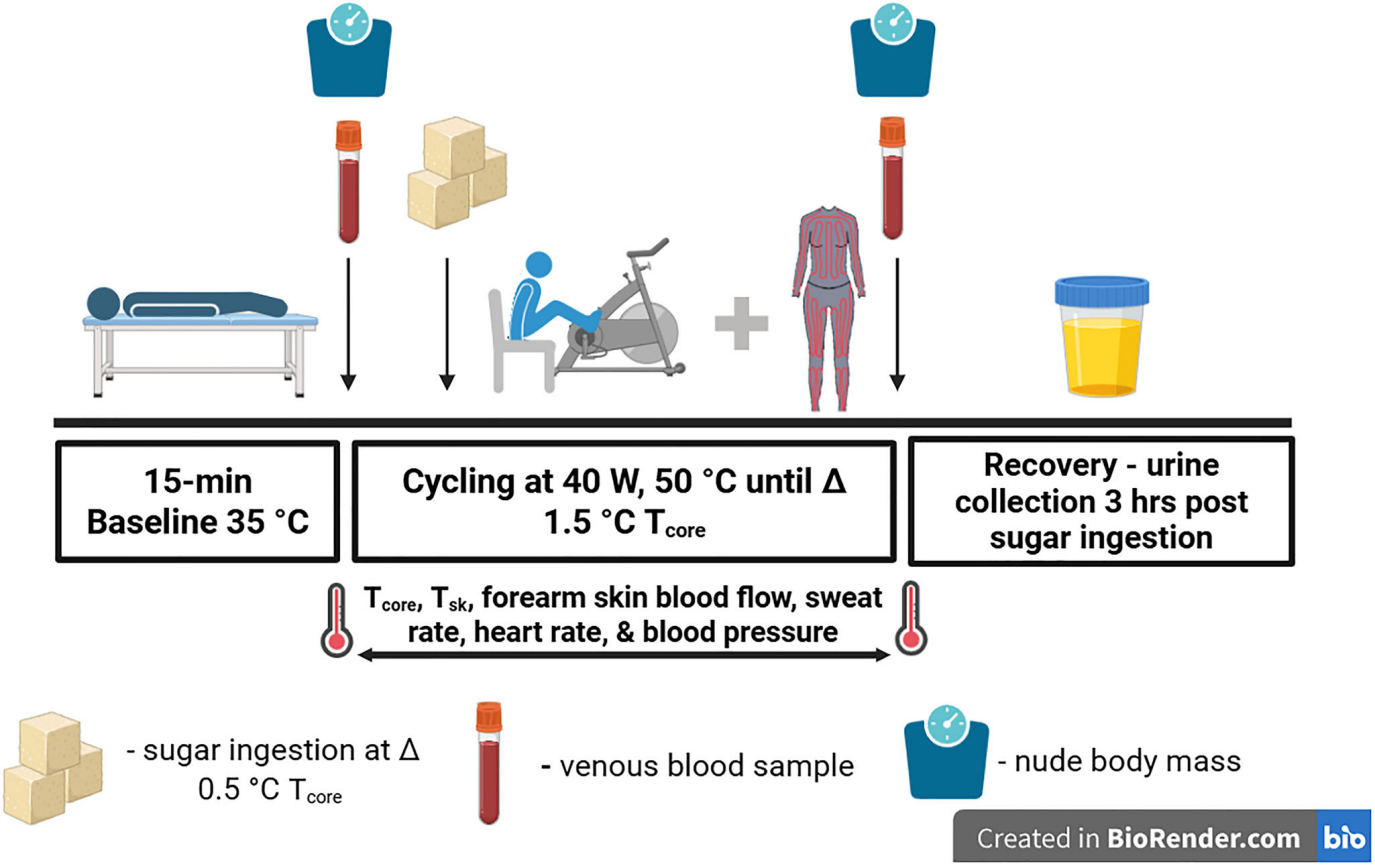
Experimental protocol of hyperthermia trial. Abbreviations: *T*_core_, core body temperature; *T*_sk_, skin temperature.

### 2.7 Blood sampling and analysis

Thirty millilitres of venous blood was obtained from the antecubital region of the arm through venepuncture at the end of the baseline period and immediately after the controlled hyperthermia period. The blood was collected into EDTA, heparin, and serum vacutainers (BD Vacutainer, BD, USA). Blood samples were centrifuged (Frontier 5000 Series Multi Pro, Ohaus, USA) at 2000*g* for 20 min at 4°C to isolate the plasma/serum, and aliquots were stored at −80°C for later analyses. We measured the sex hormones oestradiol (Human Estradiol E2 ELISA kit, Invitrogen, USA) and progesterone (Human Progesterone ELISA kit, Abcam, UK) at the baseline of the hyperthermia trial.

### 2.8 Urine storage and analysis

Urine samples were stored at −80°C until analysis. The concentrations of each sugar were determined by gas chromatography–mass spectrometry (Agilent 6890N, 5973 MSD). Ten microlitres of sample, standards and controls were transferred to 1.5 mL microcentrifuge tubes and mixed with 10 µL of internal standard solution (see below), evaporated to dryness and derivatised with methoxyamine hydrochloride (in pyridine), then *N*,*O*‐bis(trimethylsilyl)trifluoroacetamide with 1% trimethylchlorosilane. Quantification was based on comparisons against the calibration curves established for each sugar in the urine sample. The chromatographic conditions used a J&W DB‐5MS UI Capillary, 30 m × 250 µm × 0.25 µm, with temperature programmed from 100°C, increasing by 20°C/min to 320°C. The final temperature was held for 4 min. Analytes were detected by selected ion monitoring (SIM), using *m*/*z* 308, 277, 437, 361 and 308 ions to quantify erythritol, rhamnose, sucrose, lactulose and sucralose, respectively. *m*/*z* 323 and 455 were chosen to measure glucose‐^13^C_6_ and sucrose‐d_6_ used as internal standards. Excellent linearity was obtained for all analytes: rhamnose (10–1000 µg/mL, *R* ^2 ^= 0.9970), lactulose (1–100 µg/mL, *R* ^2 ^= 0.9962) and sucrose (1–100 µg/mL, *R* ^2 ^= 0.9990). The amount of each ingested sugar was determined by multiplying the measured concentration of each sugar by the total volume of urine collected and dividing by the dose administered.

### 2.9 Data analysis

The *T*_sk_, heart rate, and forearm skin blood flow data were extracted by Lab Chart software (Lab Chart Pro, ADInstruments, New Zealand), converted into 1 min averages and taken at 5 min intervals for data analysis. The *T*_core_ was sampled every 30 s and is reported in 5 min intervals. Blood pressure was measured at 5 min intervals during baseline and every 15 min during the controlled hyperthermia period. Whole‐body sweat loss was calculated as the difference in dry nude body mass before and after heating, after correcting for the volume of fluid in the multi‐sugar solution (100 mL). Mean arterial pressure (MAP) was calculated as: (⅓ × diastolic blood pressure) + (⅔ × systolic blood pressure). The *T*_sk_ was calculated as: (chest *T*_sk_ + triceps *T*_sk_) × 0.3 + (quadricep *T*_sk_ + calf *T*_sk_) × 0.2 (Ramanathan, 1964). Cutaneous vascular conductance (CVC) was calculated as: Flow (perfusion units)/MAP. The CVC was then normalised as a percentage of maximum (Chaseling et al., 2020). Body surface area was calculated using the equation defined by Du Bois and Du Bois (1989). Lower sample sizes were reported in menstrual phase hormone data owing to difficulties of obtaining venous blood samples (*n* = 5). Likewise, difficulties obtaining a maximum CVC response resulted in a lower sample size in forearm CVC data (*n* = 7).

### 2.10 Statistical analyses

A priori power analysis was not conducted owing to the limited and equivocal research investigating sex differences in gastrointestinal permeability during heat stress. The target of 24 participants was informed by previous studies investigating sex differences (male *n* = 9, female *n* = 10, Rubio et al., 2021; male *n* = 13, females *n* = 11, Snipe & Costa., 2018) and age differences (younger *n* = 9, older *n* = 9, McKenna et al., 2024) in gastrointestinal permeability. Although previous research has reported inconsistent effects of sex on gastrointestinal permeability, none has examined associations between gastrointestinal permeability and menstrual cycle hormones. Therefore, based on sample sizes from limited research and funding constraints, a total of 24 participants was considered appropriate. Statistical analyses were performed using Prism v.11 (GraphPad software, Dotmatics, USA). Data were inspected for normal distribution via visual inspection of qq plots. Participant characteristics were compared between males and females using Student's unpaired *t*‐test or the Mann–Whitney *U*‐test (height and ${\dot{V}}_{O_{2}\max}$). We analysed the effects of sex using a two‐way mixed model, with main effects of time and sex group. The number of time levels depended on whether we assessed gastrointestinal permeability (two levels: control vs. hyperthermia) or physiological variables (four levels: Δ 0, 0.5, 1.0, 1.5°C), and sex included two levels (males vs. females). The time taken for participants to reach +1.5°C *T*_core_ was analysed using Kaplan–Meier survival curves. Differences between males and females to reach this point were assessed using the log‐rank test.

As an exploratory analysis, Pearson's *r* correlations were used to determine whether morphological and thermoregulatory factors were linked with the time to reach 1.5°C *T*_core_. Pearson's *r* or Spearman rank correlations (depending on normality) were used to determine whether the change in markers of gastrointestinal barrier permeability were related to thermoregulatory (body mass loss percentage and rate of rise *T*_core_) and menstrual phase hormones (oestradiol and progesterone). Benjamini–Hochberg corrections were applied to control the false discovery rate when performing multiple correlations (Benjamini & Hochberg, 1995). Correlations were grouped into prespecified families according to the physiological outcome of interest (heating time, Δ L/R ratio, and Δ sucrose recovery correlations). Given that menstrual cycle hormones were not measured during the control trial, correlations of absolute L/R and sucrose recovery during the hyperthermia trial alone are available in the Appendix (Figures A1 and A2). In addition, correlations were performed between absolute L/R and sucrose recovery during the hyperthermia trial with the progesterone/oestradiol ratio (Figures A1 and A2). Parametric data are reported as the mean ± SD, and non‐parametric data are reported as the median (interquartile range). Statistical significance was accepted at the *P* < 0.05 level. When the *P*‐value was borderline (between 0.05 and 0.1), we show the Cohen's d for paired samples effect size ($d_{z}$) and 95% confidence interval (CI) of the mean to aid in interpretation (ΔL/R).

## 3 RESULTS

### 3.1 Thermoregulatory, cardiovascular and perceptual responses to hyperthermia

#### 3.1.1 Effects of heat

Hyperthermia combined with light cycling resulted in the expected increases in *T*_core_, *T*_sk_, mean body temperature (*T*_body_), heart rate, forearm CVC, rate–pressure product (RPP), thermal sensation, thermal comfort and rating of perceived exertion (time *P* < 0.001; Figure 2). However, there was no difference in mean arterial pressure from pre‐ to post‐trial [ΔMAP = −4 ± 14 mmHg (95% CI −10 to 2), time *P* = 0.072, $d_{z}$ = 0.29].

**FIGURE 2:**
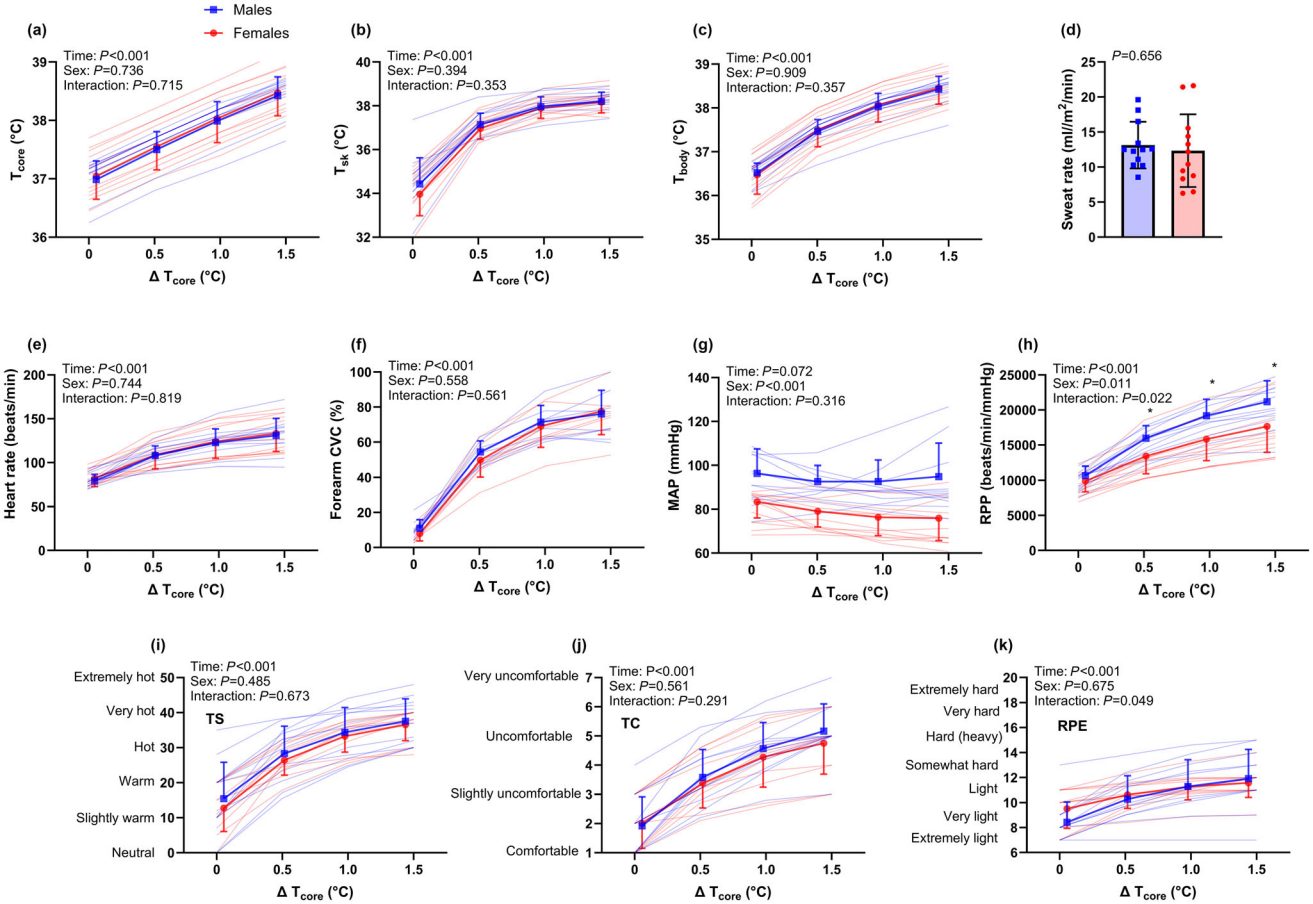
Physiological responses during hyperthermia trial: (a) absolute core body temperature (*T*_core_); (b) mean skin temperature (*T*_sk_); (c) mean body temperature (*T*_body_); (d) sweat rate; (e) heart rate; (f) forearm cutaneous vascular conductance (CVC); (g) mean arterial pressure (MAP); (h) rate–pressure product; (i) thermal sensation; (j) thermal comfort; and (k) rating of perceived exertion (RPE). Responses are shown in male (*n* = 12) and female (*n* = 12) participants except within forearm CVC; males (*n* = 9), females (*n* = 8). Blue squares/lines represent the male group, and red circles/lines represent the female group. Thick lines denote group means and thin lines denote individuals. *Significant difference between male and female groups (*P* < 0.05). Values are presented as the mean ± SD.

#### 3.1.2 Effects of sex on responses to heat

There was no difference in time to reach Δ1.5°C *T*_core_ between males and females (*P* = 0.372; Figure 3a). Time to reach Δ1.5°C *T*_core_ was correlated with body mass (*r* = 0.475, *P* = 0.057), mass‐specific surface area (*r* = −0.459, *P* = 0.036) and baseline *T*_core_ (*r* = 0.413, *P* = 0.045; Figure 3). Additional thermoregulatory characteristics compared between males and females are presented in Table 2. However, MAP responded differently between the sexes. Throughout the heating, females on average had a lower MAP compared with males (76 ± 9 vs. 92 ± 11 mmHg, group *P* < 0.001), but no interaction between time and sex was observed (interaction *P* = 0.357). Throughout the heating protocol, females had, on average, a lower RPP compared with males (13381 ± 4007 vs. 15926 ± 4553 beats/min/mmHg, group *P* = 0.011). At baseline there were no differences in RPP, but females showed a lower RPP throughout heating (interaction *P* = 0.022), such that RPP was lower in females at Δ0.5°C *T*_core_ (Δ2561 ± 2099 beats/min/mmHg, *P* = 0.009), Δ1.0°C *T*_core_ (Δ3356 ± 2534 beats/min/mmHg, *P* = 0.007) and Δ1.5°C *T*_core_ (Δ3502 ± 3539 beats/min/mmHg, *P* = 0.019). There were no other interaction effects between sex and time.

**FIGURE 3:**
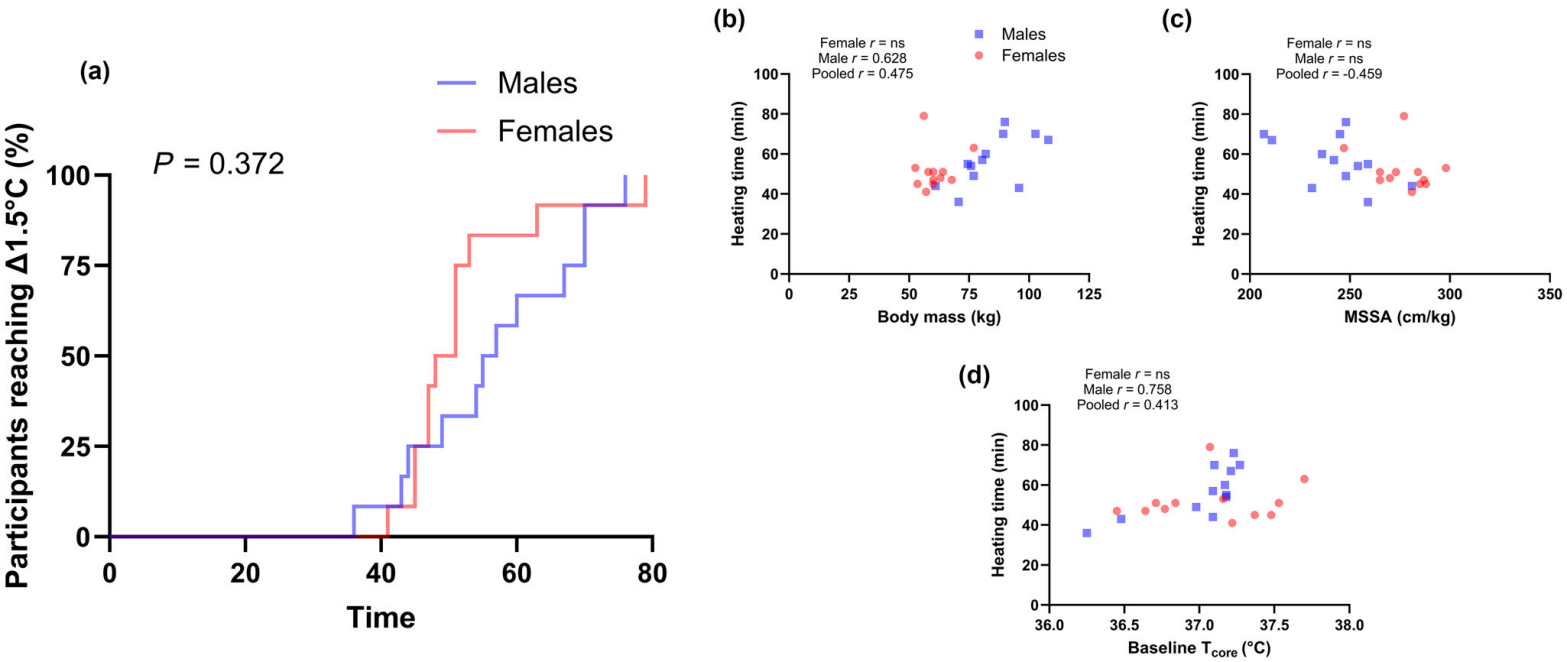
(a) Kaplan–Meier curve displaying time taken for participants to reach 1.5°C core body temperature. (b–d) Correlation analyses between heating time of participants and their body and thermoregulatory characteristics: (b) body mass; (c) mass‐specific surface area (MSSA); and (d) baseline core body temperature (*T*_core_). Blue lines/squares represent the male group (*n* = 12), and red lines/circles represent the female group (*n* = 12).

**TABLE 2:** Thermoregulatory characteristics.

CharacteristicZ	Males	Females	*P*‐value
Heating time, min	57 ± 12	52 ± 10	0.292
WBSL, mL	1389 ± 447	924 ± 356	0.010^*^
WBSL, mL/m^2^	683 ± 187	551 ± 205	0.115
AUC for body temperature, °C/min	35 ± 8	32 ± 6	0.382

*Note*: Values are presented as the mean ± SD. Males, *n* = 12. Females, *n* = 12.

Abbreviations: AUC, area under the curve; WBSL, whole body sweat loss.

*Significant difference between males and females (*P* < 0.05).

### 3.2 Markers of gastrointestinal permeability

#### 3.2.1 Effects of heat

Overall, hyperthermia combined with light cycling resulted in increases in the L/R ratio (0.062 ± 0.020) compared with the control (0.050 ± 0.019) trials [ΔL/R = 0.012 ± 0.029 (95% CI 0.000 to 0.025), *P* = 0.050, $d_{z}$ = 0.43; Figure 4]. However, there were no differences observed in urinary sucrose recovery between the control (0.029% ± 0.018 %) and hyperthermia (0.043% ± 0.044 %) trials (time *P* = 0.156).

**FIGURE 4:**
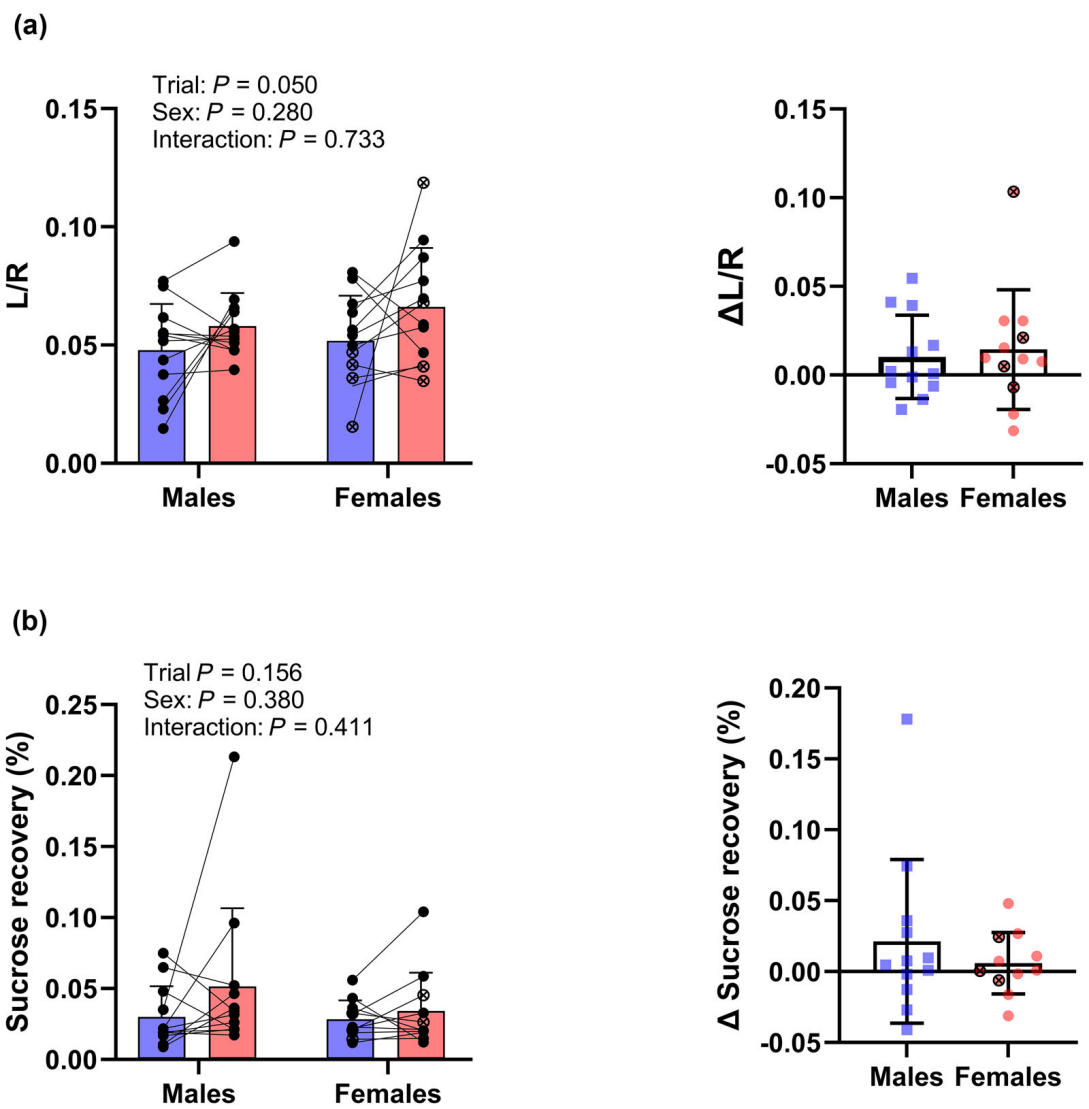
Gastrointestinal permeability was assessed between control and hyperthermia trials within males (*n* = 12) and females (*n* = 12). (a) The lactulose‐to‐rhamnose (L/R) ratio was a marker of small intestinal permeability. (b) Sucrose recovery was a marker of gastroduodenal permeability. Graphs on the right of each panel show the change between control and hyperthermia trials. Values are presented as the mean ± SD. *n* = 24. Cross‐hatches represent females on oral contraceptive (*n* = 4).

#### 3.2.2 Effects of sex on responses to heat

Across the control and hyperthermia trials, there were no differences in the ΔL/R ratio at the group level between males (0.048 ± 0.020 vs. 0.058 ± 0.014) and females (0.052 ± 0.019 vs. 0.066 ± 0.025) (group *P* = 0.733; Figure 4a). There were also no differences at the group level in urinary sucrose recovery between males (0.030% ± 0.022% vs. 0.051% ± 0.055%) and females (0.028% ± 0.013% vs. 0.034% ± 0.027%) (group *P* = 0.411; Figure 4b).

### 3.3 Correlation analyses

The ΔL/R was positively correlated with percentage body mass loss (*r* = 0.570, *P* = 0.011; Figure 5a). Within females specifically, ΔL/R showed a strong positive correlation with baseline oestradiol concentrations (*r* = 0.709, *P* = 0.040; Figure 5b), but not with baseline progesterone concentrations (*r* = −0.188, *P* = 0.607; Figure 5c).

**FIGURE 5:**
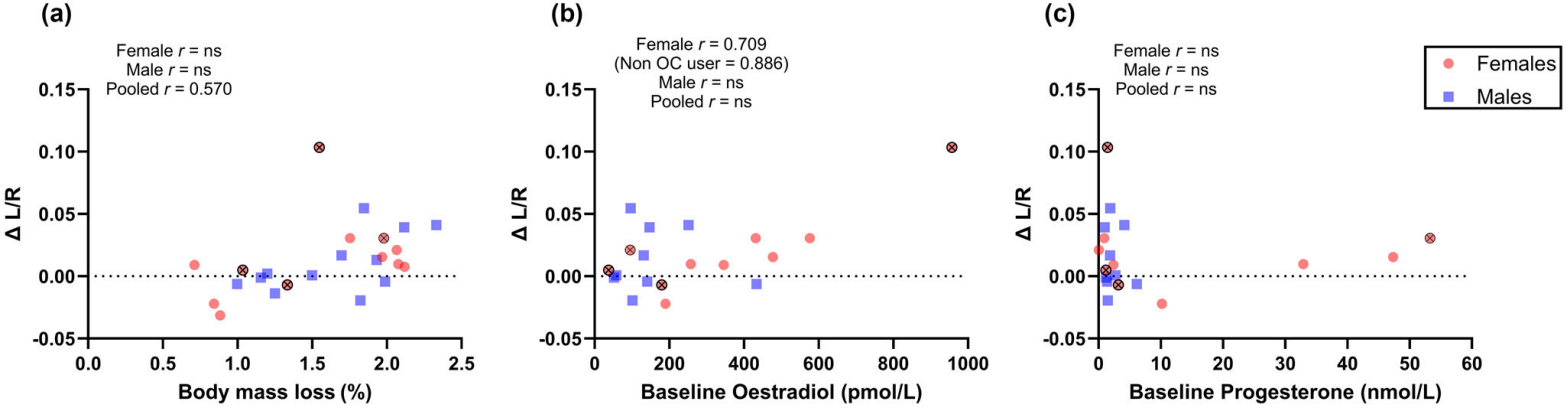
Correlation analyses between the change in the lactulose‐to‐rhamnose ratio (L/R) and thermoregulatory and plasma variables: (a) percentage body mass loss; (b) baseline oestradiol concentrations (*n* = 19, 9 males and 10 females), analysis following removal of 956 pmol/L participant (*r* = 0.600, *P* = 0.096); and (c) baseline progesterone concentrations (*n* = 19, 9 males and 10 females). Blue squares represent the male group, and red circles represent the female group. Cross‐hatches represent females on oral contraceptive (*n* = 4).

The Δ sucrose recovery was positively correlated with the rate of rise *T*_core_ (*r* = 0.608, *P* = 0.006). However, there was no correlation between Δ sucrose recovery and either baseline oestradiol (*r* = −0.150, *P* = 1.000) or baseline progesterone concentrations (*r* = −0.033, *P* = 0.948) within females (Figure 6).

**FIGURE 6:**
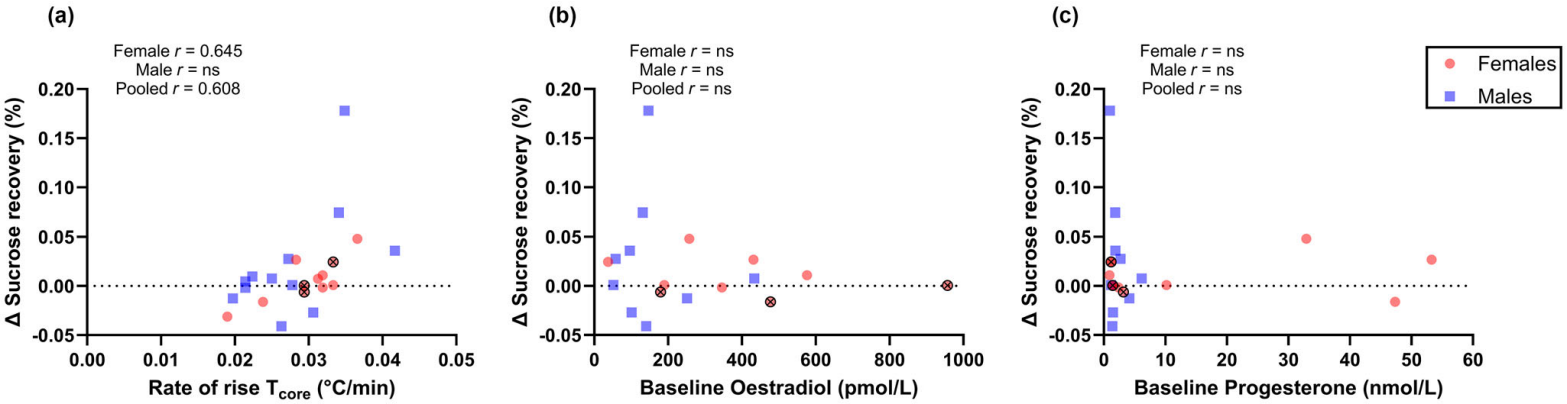
Correlation analyses between the change in sucrose recovery and thermoregulatory and plasma variables: (a) rate of rise of core temperature (*T*_core_); (b) baseline oestradiol concentrations (*n* = 18, 9 males and 9 females); and (c) baseline progesterone concentrations (*n* = 18, 9 males and 9 females). Blue squares represent the male group; red circles represent the female group. Cross‐hatches represent females on oral contraceptive (*n* = 3).

## 4 DISCUSSION

The present study aimed to investigate the effect of sex on gastrointestinal permeability during UHS. Sex differences were compared using a model of controlled hyperthermia (1.5°C increase in *T*_core_). Initially, the findings indicated an increase in markers of small intestinal permeability but no differences in gastroduodenal permeability. Although no sex differences were noted at the group level, we documented a strong positive correlation between baseline oestradiol concentrations and change in small intestinal permeability between control and hyperthermia trials (ΔL/R, *r* = 0.709).

Broadly, there were minimal differences in psychophysiological responses between the males and females. For example, core and skin temperature, heart rate, and thermal perception were not different between the sexes (Figure 2). Furthermore, we show using Kaplan–Meier survival curves that the time to reach +1.5°C was not different (Figure 3; *P* = 0.372). Such findings were expected a priori because the uncompensable nature of the heating protocol (impermeable water‐perfused suit) does not allow for potential sex differences in sweat rate (Gagnon & Kenny, 2012) to modify evaporative cooling. Predictors that were correlated with heating time during UHS were body mass (*r* = 0.475, *P* = 0.057), mass‐specific surface area (*r *= −0.459, *P* = 0.036) and baseline *T*_core_ (*r *= 0.413, *P* = 0.045; Figure 3). During UHS, the slightly accelerated heating time in females is therefore likely to be a by‐product of their body characteristics, as shown previously (Havenith & van Middendorp, 1990). We report a consistently lower MAP in females at baseline and throughout the heating period (Figure 2g), probably attributable to menstrual phase hormones and known sex differences in autonomic regulation of blood pressure (Hart & Charkoudian, 2014).

The lack of difference in ΔL/R at the group level aligns with previous research during running in the heat (Snipe & Costa, 2018). However, L/R ratio was measured only during an exertional trial without corresponding control conditions, meaning that they were unable to determine whether hyperthermia changed permeability relative to control conditions. In contrast, Rubio et al. (2021) reported a lower ΔL/R ratio in females compared with males following prolonged sauna exposure with dehydration. However, similarly to our study, they observed no sex differences in gastroduodenal permeability measured via sucrose recovery. Interestingly, the absolute ΔL/R in both previous studies was greater than shown in our data, which was probably attributable to a greater level of thermal strain, as suggested previously (Pires et al., 2017). Owing to the large variability in ΔL/R responses within the female group, although there was no sex difference at the group level, such analysis alone is likely to mask a potential hormone‐dependent subgroup effect that might be driving the female individual variability observed.

Despite a lack of difference at the group level, we document a novel positive correlation between baseline oestradiol concentrations and ΔL/R ratio (*r* = 0.709, *P* = 0.040). Although the precise mechanism for the correlation remains unclear, it might involve stress‐induced activation of colonic mast cells. In rodents, activation of colonic mast cells is strongly linked to gastrointestinal barrier disruption, and importantly, oestrogen potentiates this response (Xu et al., 2020). Consequently, greater systemic quantities of oestrogen might impair gastrointestinal barrier function through actions on colonic mast cells. In humans, increases in gastrointestinal permeability were abolished following the administration of mast cell stabilizers (Vanuytsel et al., 2014), lending support for this pathway. Further support is provided by clinical populations in which mast cell stabilizers improved gastrointestinal symptoms in patients with irritable bowel syndrome (Lobo et al., 2017). Oestradiol may also play a direct role in the regulation of tight junctions, which connect the intestinal enterocyte cells at the barrier and regulate paracellular permeability (König et al., 2016). However, research suggests varying roles of oestradiol in tight junction protein function, demonstrating either impaired (Zhou et al., 2017) or improved (Song et al., 2018) function. Therefore, more evidence is needed to determine the precise impact of oestradiol on heat‐induced gastrointestinal permeability in humans.

Our study showed that a +1.5°C increase in *T*_core_ caused a modest increase in L/R compared with control conditions. The direction of this effect is consistent with previous research demonstrating an elevated ΔL/R in response to heat exposure (Houghton et al., 2023; McKenna et al., 2024; Pugh et al., 2017; Rubio et al., 2021; Zuhl et al., 2015). Pooling all participants, the ΔL/R also appears to be linked to sweat loss (percentage body mass loss), because the present study found that percentage body mass loss was positively correlated with ΔL/R (*r* = 0.570, *P* = 0.011). Hypohydration resulting in a 3% body mass loss has been shown to compromise gastrointestinal barrier integrity to a greater extent than euhydration during exercise in young males (Costa et al., 2019). Although the exact mechanisms remain unclear, it is likely that dehydration exacerbates gastrointestinal permeability by reducing blood volume, further diminishing splanchnic blood flow (Ter Steege & Kolkman, 2012). The pathway supports the finding in the present study that individuals with greater body mass loss, and probably greater blood volume loss, experienced greater small intestinal permeability. The detection of an increase in small intestinal permeability during a relatively short‐term UHS exposure (54 ± 11 min) is likely to be conservative in comparison to the more prolonged UHS exposures experienced by heat‐vulnerable occupations. Real‐world shift patterns might show exacerbated gastrointestinal barrier permeability.

In contrast to the small intestinal findings, heat did not impact urinary sucrose recovery in our protocol. These findings align with the findings of McKenna et al. (2024), who reported no change in sucrose recovery in young adults, despite a higher *T*_core_ increase of +2.0°C from baseline. However, these results contrast with those of Rubio et al. (2021), who observed an increase in urinary sucrose recovery following sauna exposure compared with control. The discrepancy might be explained by differences in the duration of thermal strain. Rubio et al. (2021) used a prolonged passive heating protocol averaging 209 ± 39 min, whereas the heating duration of the present study (54 ± 11 min) and that of McKenna et al. (2024) (72 ± 4 min) were considerably shorter. Furthermore, it is possible that those in the study by Rubio et al. (2021) might have had higher *T*_core_, as suggested by their tympanic temperature data, which are difficult to validate. Nevertheless, an increase in small intestinal permeability in the absence of a change in gastroduodenal permeability is consistent with high‐intensity exercise research (Davis et al., 2005; Pals et al., 1997), suggesting that, in young adults, the gastroduodenal barrier might have greater resilience to hypoxia than the small intestine. This resilience could potentially be attributed to the robust, two‐layer mucus barrier in the stomach, which contrasts with the single, less‐adherent mucus layer found in the small intestine (Atuma et al., 2001).

### 4.1 Limitations

There are limitations that should be considered when interpreting our data. First, the water‐perfused suit model induces UHS that matched *T*_core_ increases across participants (i.e., +1.5°C). This approach might not capture potentially divergent body temperature patterns between the sexes during real‐world ambient heat exposures. However, this model allows for the isolation of sex‐specific effects on gastrointestinal barrier responses to matched hyperthermia.

Second, exercise alone has been shown to increase gastrointestinal barrier permeability (Davis et al., 2005). Therefore, the design of our study is unable to attribute the effects observed entirely to heat stress per se. However, research suggests a level of high exercise intensity is required toincrease gastrointestinal permeability, with low‐to‐moderate exercise intensity showing no change in the L/R ratio (Pals et al., 1997). In addition to this, trial order was fixed such that the control trial always preceded the hyperthermia trial. Although this approach was taken to familiarise participants to new laboratory settings prior to the heat trial, this design cannot rule out the possibility of order effects.

Third, although the present study recorded menstrual phase and female hormone concentrations (oestradiol and progesterone), it did not control for these factors. Menstrual phase has known impacts on thermoregulation (Charkoudian & Stachenfeld, 2014; Kenshalo, 1966) and may impact resting gastrointestinal barrier integrity (Flood et al., 2022). However, by not restricting the female cohort to a single phase, we were able to capture a diverse range of hormone concentrations, ultimately facilitating the detection of a strong positive correlation between circulating oestradiol and gastrointestinal barrier permeability. It is worth noting that one oral contraceptive user reported greater oestradiol concentrations than would be expected with daily use (956 pmol/L); however, we obtained insufficient data on contraceptive use frequency and dose to clarify these findings. Importantly, when this participant was removed from the analysis, the correlation between baseline oestradiol concentrations and ΔL/R was reduced from strong to moderate (*r* = 0.600, *P* = 0.096), indicating that the individual with high plasma oestradiol concentrations had an impact on the correlation. Future research should investigate the role of menstrual phase hormones on heat‐induced gastrointestinal barrier permeability in a larger sample size and better controlling for oral contraceptive use. The present study did not assess large intestine permeability, which houses the greatest concentration of bacteria in the gastrointestinal tract (Dieterich et al., 2018) and will probably be a major source of microbial translocation preceding endotoxaemia.

Lastly, the participants recruited for the study were young and healthy, meaning that our findings cannot readily be extrapolated to intrinsically heat‐vulnerable occupations.

## 5 CONCLUSION

In conclusion, our study found a modest increase in small intestinal permeability during UHS in young adults but no differences in gastrointestinal permeability between sexes at the group level. However, we show for the first time that the extent of rise of small intestinal permeability during UHS was strongly correlated with baseline oestradiol concentrations within females. Future studies should confirm whether oestradiol plays an independent role in modulating heat‐induced gastrointestinal permeability in humans.

## AUTHOR CONTRIBUTIONS

Josh Foster, Shoji Leach and Stephen D.R. Harridge conceived and designed the study. Shoji Leach, Ellie J. Allsop, Isobel A. Martin and Josh Foster performed the experiments. Shoji Leach, Thomas Francis, John M. Halket, Ana Rodriguez‐Mateos and Anna Caldwell performed biochemical assays and analysis. Nabeel Sheikh assisted with participant screening and 12‐lead ECG interpretation. Shoji Leach drafted the manuscript. All authors interpreted the results of experiments. Shoji Leach prepared figures. All authors edited and revised the manuscript. All authors approved the final version of the manuscript and agree to be accountable for all aspects of the work in ensuring that questions related to the accuracy or integrity of any part of the work are appropriately investigated and resolved. All persons designated as authors qualify for authorship, and all those who qualify for authorship are listed.

## CONFLICT OF INTEREST

The authors have no conflicts of interest to declare.

## GENERATIVE AI STATEMENT

No generative AI tools were used in the preparation of this manuscript.

## Figures and Tables

**FIGURE A1:**
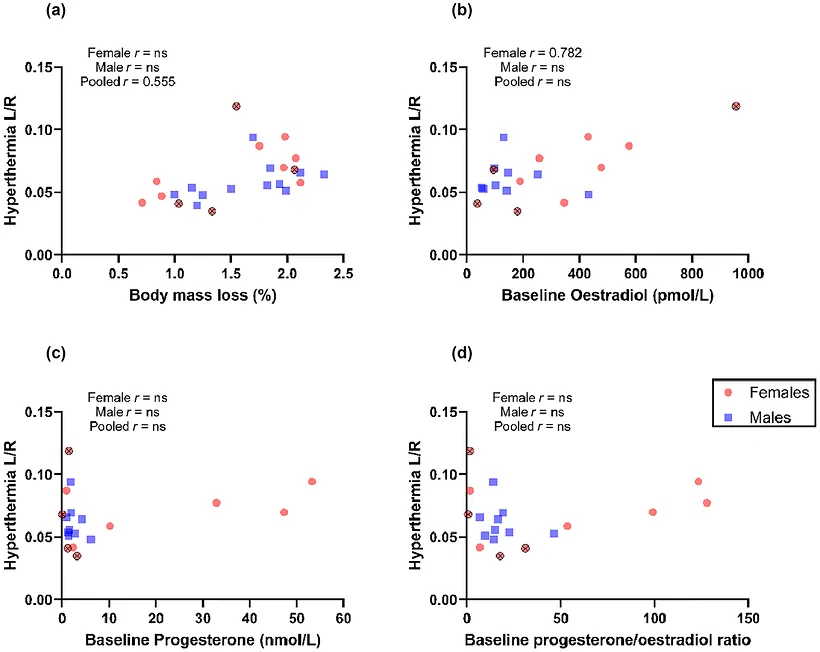
Correlation analyses between the lactulose‐to‐rhamnose ratio (L/R) during the hyperthermia trial and thermoregulatory and plasma variables: (a) percentage body mass loss (*P* = 0.005); (b) baseline oestradiol concentrations (*P* = 0.011) (*n* = 19, 9 males and 10 females); (c) baseline progesterone concentrations (*n* = 19, 9 males and 9 females); and (d) baseline progesterone/oestradiol ratio (*n* = 18, 9 males and 9 females). Cross‐hatches represent females on oral contraceptive (*n* = 4).

**FIGURE A2:**
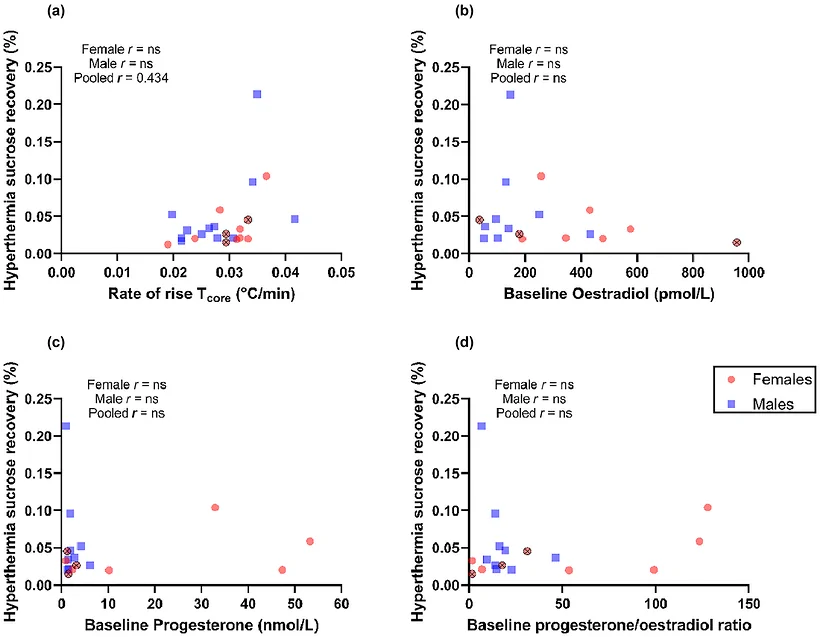
Correlation analyses between sucrose recovery during the hyperthermia trial and thermoregulatory and plasma variables: (a) rate of rise of core body temperature (*T*_core_; *P* = 0.038); (b) baseline oestradiol concentrations (*n* = 18, 9 males and 9 females); (c) baseline progesterone concentrations (*n* = 18, 9 males and 9 females); and (d) baseline progesterone/oestradiol ratio (*n* = 18, 9 males and 9 females). Cross‐hatches represent females on oral contraceptive (*n* = 3).

## Data Availability

Data are available upon reasonable request to the corresponding author (J.F.).

## References

[eph70558-bib-0001] Atuma, C. , Strugala, V. , Allen, A. , & Holm, L. (2001). The adherent gastrointestinal mucus gel layer: Thickness and physical state in vivo. American Journal of Physiology—Gastrointestinal and Liver Physiology, 280(5), 43–45. 10.1152/ajpgi.2001.280.5.G922 11292601

[eph70558-bib-0002] Benjamini, Y. , & Hochberg, Y. (1995). Controlling the False Discovery Rate: A Practical and Powerful Approach to Multiple Testing. Journal of the Royal Statistical Society Series B: Statistical Methodology, 57(1), 289–300.

[eph70558-bib-0051] Borg, G. (1970). Perceived exertion as an indicator of somatic stress. Journal of Rehabilitation Medicine, 2(2), 92–98. 5523831

[eph70558-bib-0003] Bongers, C. C. W. G. , de Korte, J. Q. , Zwartkruis, M. , Levels, K. , Kingma, B. R. M. , & Eijsvogels, T. M. H. (2022). Heat Strain and Use of Heat Mitigation Strategies Among COVID‐19 Healthcare Workers Wearing Personal Protective Equipment—A Retrospective Study. International Journal of Environmental Research and Public Health, 19(3), 1905. 10.3390/ijerph19031905 PMC8834922 35162925

[eph70558-bib-0004] Camilleri, M. (2021). Human intestinal barrier: Effects of stressors, diet, prebiotics, and probiotics. Clinical and Translational Gastroenterology, 12(1), e00308. 10.14309/ctg.0000000000000308 PMC7838004 33492118

[eph70558-bib-0005] Charkoudian, N. , & Stachenfeld, N. S. (2014). Reproductive hormone influences on thermoregulation in women. Comprehensive Physiology, 4(2), 793–804. 10.1002/cphy.c130029 24715568

[eph70558-bib-0006] Chaseling, G. K. , Crandall, C. G. , & Gagnon, D. (2020). Skin blood flow measurements during heat stress: Technical and analytical considerations. American Journal of Physiology—Regulatory Integrative and Comparative Physiology, 318(1), R57–R69. 10.1152/ajpregu.00177.2019 31596109

[eph70558-bib-0007] Cheung, S. S. , McLellan, T. M. , & Tenaglia, S. (2000). The thermophysiology of uncompensable heat stress: Physiological manipulations and individual characteristics. Sports Medicine, 29(5), 329–359. 10.2165/00007256-200029050-00004 10840867

[eph70558-bib-0008] Costa, R. J. S. , Camões‐Costa, V. , Snipe, R. M. J. , Dixon, D. , Russo, I. , & Huschtscha, Z. (2019). Impact of exercise‐induced hypohydration on gastrointestinal integrity, function, symptoms, and systemic endotoxin and inflammatory profile. Journal of Applied Physiology, 126(5), 1281–1291. 10.1152/japplphysiol.01032.2018 30896356

[eph70558-bib-0009] Davis, M. S. , Willard, M. D. , Williamson, K. K. , Steiner, J. M. , & Williams, D. A. (2005). Sustained strenuous exercise increases intestinal permeability in racing Alaskan sled dogs. Journal of Veterinary Internal Medicine, 19(1), 34–39. 10.1892/0891-6640(2005)19<34:sseiip>2.0.co;2 15715045

[eph70558-bib-0010] Dieterich, W. , Schink, M. , & Zopf, Y. (2018). Microbiota in the Gastrointestinal Tract. Medical sciences. (Basel, Switzerland), 6(4), 116. 10.3390/medsci6040116 PMC6313343 30558253

[eph70558-bib-0011] Du Bois, D. , & Du Bois, E. F. (1989). A formula to estimate the approximate surface area if height and weight be known. 1916. Nutrition, (Burbank, Los Angeles County, Calif.), 5(5), 303–313. 2520314

[eph70558-bib-0012] Duncan, H. W. , Gardner, G. W. , & James Barnard, R. (1979). Physiological Responses of Men Working in Fire Fighting Equipment in the Heat. Ergonomics, 22(5), 521–527. 10.1080/00140137908924636 520284

[eph70558-bib-0013] Flood, T. R. , Kuennen, M. R. , Blacker, S. D. , Myers, S. D. , Walker, E. F. , & Lee, B. J. (2022). The effect of sex, menstrual cycle phase and oral contraceptive use on intestinal permeability and ex‐vivo monocyte TNFα release following treatment with lipopolysaccharide and hyperthermia. Cytokine, 158, 155991. 10.1016/j.cyto.2022.155991 35944412

[eph70558-bib-0014] Gagnon, D. , & Kenny, G. P. (2012). Sex differences in thermoeffector responses during exercise at fixed requirements for heat loss. Journal of Applied Physiology, 113(5), 746–757. 10.1152/japplphysiol.00637.2012 22797311

[eph70558-bib-0015] Golding, L. , & Sinning, W. (1989). Y's Way to physical fitness: The complete guide to fitness testing and instruction: YMCA of the USA. Human Kinetics Publishers.

[eph70558-bib-0016] Hart, E. C. J. , & Charkoudian, N. (2014). Sympathetic neural regulation of blood pressure: Influences of sex and aging. Physiology, 29(1), 8–15. 10.1152/physiol.00031.2013 24382867

[eph70558-bib-0017] Havenith, G. , Luttikholt, V. G. M. , & Vrijkotte, T. G. M. (1995). The relative influence of body characteristics on humid heat stress response. European Journal of Applied Physiology and Occupational Physiology, 70(3), 270–279. 10.1007/BF00238575 7607204

[eph70558-bib-0018] Havenith, G. , & van Middendorp, H. (1990). The relative influence of physical fitness, acclimatization state, anthropometric measures and gender on individual reactions to heat stress. European Journal of Applied Physiology and Occupational Physiology, 61(5–6), 419–427. 10.1007/BF00236062 2079061

[eph70558-bib-0019] Hooper, L. V. , & MacPherson, A. J. (2010). Immune adaptations that maintain homeostasis with the intestinal microbiota. In Nature Reviews Immunology, 10(3), 159–169. 10.1038/nri2710 20182457

[eph70558-bib-0020] Houghton, M. J. , Snipe, R. M. J. , Williamson, G. , & Costa, R. J. S. (2023). Plasma measurements of the dual sugar test reveal carbohydrate immediately alleviates intestinal permeability caused by exertional heat stress. Journal of Physiology, 601(20), 4573–4589. 10.1113/JP284536 37695123

[eph70558-bib-0021] ISO10551 . (2001). Ergonomics of the thermal environment — assessment of the influence of the thermal environment using subjective judgement scales.

[eph70558-bib-0022] Jegodka, Y. , Lagally, L. , Mertes, H. , Deering, K. , Schoierer, J. , Buchberger, B. , & Bose‐O'Reilly, S. (2021). Hot days and Covid‐19: Online survey of nurses and nursing assistants to assess occupational heat stress in Germany during summer 2020. Journal of Climate Change and Health, 3, 100031. 10.1016/j.joclim.2021.100031 PMC8925298 35313705

[eph70558-bib-0023] Kenshalo, D. R. (1966). Changes in the cool threshold associated with phases of the menstrual cycle. Journal of Applied Physiology, 21(3), 1031–1039. 10.1152/jappl.1966.21.3.1031 5912720

[eph70558-bib-0024] König, J. , Wells, J. , Cani, P. D. , García‐Ródenas, C. L. , MacDonald, T. , Mercenier, A. , Whyte, J. , Troost, F. , & Brummer, R. J. (2016). Human intestinal barrier function in health and disease. Clinical and Translational Gastroenterology, 7(10), e196. 10.1038/ctg.2016.54 PMC5288588 27763627

[eph70558-bib-0025] Leon, L. R. , & Helwig, B. G. (2010). Heat stroke: Role of the systemic inflammatory response. Journal of Applied Physiology, 109(6), 1980–1988. 10.1152/japplphysiol.00301.2010 20522730

[eph70558-bib-0026] Lobo, B. , Ramos, L. , Martínez, C. , Guilarte, M. , González‐Castro, A. M. , Alonso‐Cotoner, C. , Pigrau, M. , de Torres, I. , Rodiño‐Janeiro, B. K. , Salvo‐Romero, E. , Fortea, M. , Pardo‐Camacho, C. , Guagnozzi, D. , Azpiroz, F. , Santos, J. , & Vicario, M. (2017). Downregulation of mucosal mast cell activation and immune response in diarrhoea‐irritable bowel syndrome by oral disodium cromoglycate: A pilot study. United European Gastroenterology Journal, 5(6), 887–897. 10.1177/2050640617691690 PMC5625876 29026603

[eph70558-bib-0027] McKenna, Z. J. , Atkins, W. C. , Wallace, T. , Jarrard, C. P. , Crandall, C. G. , & Foster, J. (2024). Gastrointestinal permeability and kidney injury risk during hyperthermia in young and older adults. Experimental Physiology, 110(1), 79–92. 10.1113/EP092204 PMC11689130 39417775

[eph70558-bib-0028] Ministry of Defence . (2025). UK armed forces biannual diversity statistics . GOV. https://www.gov.uk/government/statistics/uk‐armed‐forces‐biannual‐diversity‐statistics‐april‐2025

[eph70558-bib-0029] Ministry of Housing, C. and L. G . (2025). FIRE1103: Staff headcount by gender, fire and rescue authority and role . GOV. https://www.gov.uk/government/statistical‐data‐sets/fire‐statistics‐data‐tables#workforce‐and‐workforce‐diversity

[eph70558-bib-0030] Minson, C. T. , Wladkowski, S. L. , Cardell, A. F. , Pawelczyk, J. A. , & Kenney, W. L. (1998). Age alters the cardiovascular response to direct passive heating. Journal of Applied Physiology, 84(4), 1323–1332. 10.1152/jappl.1998.84.4.1323 9516200

[eph70558-bib-0031] Notley, S. R. , Meade, R. D. , & Kenny, G. P. (2021). Time following ingestion does not influence the validity of telemetry pill measurements of core temperature during exercise‐heat stress. The journal Temperature toolbox. Temperature, 8(1), 12–20. 10.1080/23328940.2020.1801119 PMC7849685 33553501

[eph70558-bib-0032] Pals, K. L. , Chang, R. T. , Ryan, A. J. , & Gisolfi, C. V. (1997). Effect of running intensity on intestinal permeability. Journal of Applied Physiology, 82(2), 571–576. 10.1152/jappl.1997.82.2.571 9049739

[eph70558-bib-0033] Pires, W. , Veneroso, C. E. , Wanner, S. P. , Pacheco, D. A. S. , Vaz, G. C. , Amorim, F. T. , Tonoli, C. , Soares, D. D. , & Coimbra, C. C. (2017). Association between exercise‐induced hyperthermia and intestinal permeability: A Systematic Review. Sports Medicine, 47(7), 1389–1403. 10.1007/s40279-016-0654-2 27943148

[eph70558-bib-0034] Powis, C. M. , Byrne, D. , Zobel, Z. , Gassert, K. N. , Lute, A. C. , & Schwalm, C. R. (2023). Observational and model evidence together support wide‐spread exposure to noncompensable heat under continued global warming. Science Advances, 9(36), eadg9297. 10.1126/sciadv.adg9297 PMC10491292 37682995

[eph70558-bib-0035] Pugh, J. N. , Sage, S. , Hutson, M. , Doran, D. A. , Fleming, S. C. , Highton, J. , Morton, J. P. , & Close, G. L. (2017). Glutamine supplementation reduces markers of intestinal permeability during running in the heat in a dose‐dependent manner. European Journal of Applied Physiology, 117(12), 2569–2577. 10.1007/s00421-017-3744-4 PMC5694515 29058112

[eph70558-bib-0036] Ramanathan, N. L. (1964). A new weighting system for mean surface temperature of the human body. Journal of Applied Physiology, 19(3), 531–533. 10.1152/jappl.1964.19.3.531 14173555

[eph70558-bib-0037] Rentería, L. I. , Zheng, X. , Valera, I. , Machin, D. R. , Leon, L. R. , & Laitano, O. (2023). Ovariectomy aggravates the pathophysiological response to exertional heat stroke in mice. Journal of Applied Physiology (Bethesda, Md. : 1985), 134(5), 1224–1231. 10.1152/japplphysiol.00092.2023 PMC10151055 37022961

[eph70558-bib-0038] Rowell, L. B. , Detry, J. R. , Profant, G. R. , & Wyss, C. (1971). Splanchnic vasoconstriction in hyperthermic man–role of falling blood pressure. Journal of Applied Physiology, 31(6), 864–869. 10.1152/jappl.1971.31.6.864 5123665

[eph70558-bib-0039] Rubio, R. F. M. , Eriksson, U. , Brummer, R. J. , & König, J. (2021). Sauna dehydration as a new physiological challenge model for intestinal barrier function. Scientific Reports, 11(1), 15514. 10.1038/s41598-021-94814-0 PMC8324874 34330970

[eph70558-bib-0040] Selkirk, G. A. , McLellan, T. M. , Wright, H. E. , & Rhind, S. G. (2008). Mild endotoxemia, NF‐κB translocation, and cytokine increase during exertional heat stress in trained and untrained individuals. American Journal of Physiology—Regulatory Integrative and Comparative Physiology, 295(2), R611–R623. 10.1152/ajpregu.00917.2007 18565834

[eph70558-bib-0041] Snipe, R. M. J. , & Costa, R. J. S. (2018). Does biological sex impact intestinal epithelial injury, small intestine permeability, gastrointestinal symptoms and systemic cytokine profile in response to exertional‐heat stress? Journal of Sports Sciences, 36(24), 2827–2835. 10.1080/02640414.2018.1478612 29790452

[eph70558-bib-0042] Song, C. H. , Kim, N. , Sohn, S. H. , Lee, S. M. , Nam, R. H. , Na, H. Y. , Lee, D. H. , & Surh, Y. J. (2018). Effects of 17β‐estradiol on colonic permeability and inflammation in an azoxymethane/dextran sulfate sodium‐induced colitis mouse model. Gut and Liver, 12(6), 682–693. 10.5009/gnl18221 PMC6254630 30400733

[eph70558-bib-0043] Ter Steege, R. W. F. , & Kolkman, J. J. (2012). Review article: The pathophysiology and management of gastrointestinal symptoms during physical exercise, and the role of splanchnic blood flow. Alimentary Pharmacology and Therapeutics, 35(5), 516–528. 10.1111/j.1365-2036.2011.04980.x 22229513

[eph70558-bib-0044] Vanuytsel, T. , van Wanrooy, S. , Vanheel, H. , Vanormelingen, C. , Verschueren, S. , Houben, E. , Salim Rasoel, S. , Tόth, J. , Holvoet, L. , Farré, R. , Van Oudenhove, L. , Boeckxstaens, G. , Verbeke, K. , & Tack, J. (2014). Psychological stress and corticotropin‐releasing hormone increase intestinal permeability in humans by a mast cell‐dependent mechanism. Gut, 63(8), 1293–1299. 10.1136/gutjnl-2013-305690 24153250

[eph70558-bib-0045] van Wijck, K. , Lenaerts, K. , Grootjans, J. , Wijnands, K. A. P. , Poeze, M. , van Loon, L. J. C. , Dejong, C. H. C. , & Buurman, W. A. (2012). Physiology and pathophysiology of splanchnic hypoperfusion and intestinal injury during exercise: Strategies for evaluation and prevention. American Journal of Physiology—Gastrointestinal and Liver Physiology, 303(2), G155–G168. 10.1152/ajpgi.00066.2012 22517770

[eph70558-bib-0046] Vecellio, D. J. , Kong, Q. , Kenney, W. L. , & Huber, M. (2023). Greatly enhanced risk to humans as a consequence of empirically determined lower moist heat stress tolerance. Proceedings of the National Academy of Sciences of the United States of America, 120(42), e2305427120. 10.1073/pnas.2305427120 PMC10589700 37812703

[eph70558-bib-0047] White, M. K. , Hodous, T. K. , & Vercruyssen, M. (1991). Effects of thermal environment and chemical protective clothing on work tolerance, physiological responses, and subjective ratings. Ergonomics, 34(4), 445–457. 10.1080/00140139108967328 1860463

[eph70558-bib-0048] Xu, S. , Wang, X. , Zhao, J. , Yang, S. , Dong, L. , & Qin, B. (2020). GPER‐mediated, oestrogen‐dependent visceral hypersensitivity in stressed rats is associated with mast cell tryptase and histamine expression. Fundamental and Clinical Pharmacology, 34(4), 433–443. 10.1111/fcp.12537 31967341

[eph70558-bib-0049] Zhou, Z. , Zhang, L. , Ding, M. , Luo, Z. , Yuan, S. , Bansal, M. B. , Gilkeson, G. , Lang, R. , & Jiang, W. (2017). Estrogen decreases tight junction protein ZO‐1 expression in human primary gut tissues. Clinical Immunology, 183, 174–180. 10.1016/j.clim.2017.08.019 PMC5673541 28867253

[eph70558-bib-0050] Zuhl, M. , Dokladny, K. , Mermier, C. , Schneider, S. , Salgado, R. , & Moseley, P. (2015). The effects of acute oral glutamine supplementation on exercise‐induced gastrointestinal permeability and heat shock protein expression in peripheral blood mononuclear cells. Cell Stress and Chaperones, 20(1), 85–93. 10.1007/s12192-014-0528-1 PMC4255255 25062931

